# PmrA Mutations in Drug-Resistant *Acinetobacter baumannii* Affect Sensor Kinase-Response Regulator Interaction and Phosphotransfer

**DOI:** 10.3390/microorganisms13112600

**Published:** 2025-11-15

**Authors:** Felicia E. Jaimes, Alexander D. Hondros, Jude Kinkead, Morgan E. Milton, Richele J. Thompson, Aimee M. Figg, Christian Melander, John Cavanagh

**Affiliations:** 1 Department of Biochemistry & Molecular Biology, Brody School of Medicine, East Carolina University, 600 Moye Blvd, Greenville, NC 27858, USA; jaimesf25@ecu.edu (F.E.J.); alexander.hondros@vanderbilt.edu (A.D.H.); kinkeadj21@students.ecu.edu (J.K.); miltonm19@ecu.edu (M.E.M.); thompsonr19@ecu.edu (R.J.T.); figga25@ecu.edu (A.M.F.); 2 Department of Chemistry and Biochemistry, University of Notre Dame, 240F McCourtney Hall–West, Notre Dame, IN 46556, USA; cmelande@nd.edu

**Keywords:** two-component systems, antibiotic resistance, response regulators, point-mutants, regulatory networks, transcriptional control

## Abstract

Multi-drug resistance in *Acinetobacter baumannii* poses a significant human health threat. For multidrug-resistant pathogens, ‘last line of defense’ antibiotics like the polymyxins are implemented. Concerningly, polymyxin-resistance is evidenced in *Acinetobacter baumannii* and is mediated by the PmrAB two-component system. The response regulator PmrA upregulates *pmrC*, leading to lipooligosaccharide modifications that reduce polymyxin binding. Sequencing of *A. baumannii* resistant isolates has identified point mutations in the receiver domain of PmrA that correlate with increased resistance. To investigate functional impacts of these mutations, we characterized five PmrA mutations (D10N, M12I, I13M, G54E, and S119T) by assessing changes in PmrA DNA-binding affinity, dimerization, phosphorylation, and structure. Our findings suggest that these mutations impact the ability of PmrA to receive the activating phosphoryl group from the sensor kinase PmrB. The slow phosphoryl uptake is likely due to (1) disruption of the PmrB-PmrA interaction by interfering with the recognition site on PmrA, or (2) perturbation of PmrA’s active site via steric hindrance or displacement of residues and ions necessary for coordination within the aspartic acid pocket. Slowed phosphorylation of a response regulator can lead to enhanced gene transcription through several mechanisms. These insights advance our understanding of PmrA-mediated resistance in *A. baumannii*.

## 1. Introduction

Antibiotic resistance is a global health crisis, projected to surpass cancer mortality rates within the coming decades [1]. Despite the clear need for new antibiotics, development has slowed due to the rapid pace of developing resistance [2]. Tackling this challenge requires a multipronged approach that addresses the complexity of the mechanisms driving resistance.

The Gram-negative pathogen *Acinetobacter baumannii* has emerged as a particularly insidious pathogen and is responsible for over one million infections globally each year, primarily in healthcare settings [3]. Most alarming is *A. baumannii*’s ability to evade polymyxins, a ‘last line of defense’ antibiotic class that is crucial in controlling this dangerous pathogen. Polymyxin usage declined in the 1970s due to associated nephrotoxicity and neurotoxicity; however, the prevalence of multidrug-resistant *A. baumannii* has caused an increase in its usage in recent years [4]. Polymyxins function by interacting with the negatively charged lipooligosaccharide (LOS) layer of *A. baumannii*, disrupting membrane integrity and causing bacterial death [5,6]. However, *A. baumannii* can modify its LOS charge to evade polymyxin action, a process primarily driven by the PmrAB two-component regulatory system [7,8]. PmrA directly upregulates the expression of the phosphoethanolamine (pEtN) transferase *pmrC*, which adds pEtN moieties to the LOS, increasing the net positive charge of LOS and repelling polymyxins [9].

Two-component systems are central to bacterial signal transduction, allowing bacteria to sense and respond to environmental changes such as nutrient shifts, pH, osmolarity, antibiotics, or host immune factors. The PmrAB two-component system consists of the histidine kinase, PmrB, which senses environmental stimuli and activates its cognate response regulator, PmrA [10]. PmrA is a two-domain protein, containing both a receiver domain (REC), which accepts a phosphoryl group from PmrB, and a DNA-binding domain (DBD). Similar to other response regulators, upon phosphorylation, PmrA dimerizes and binds to a conserved promoter sequence known as the ‘PmrA box’ [9]. PmrA-DNA binding drives the expression of genes that modify the LOS composition. Our lab previously identified the *A. baumannii*-specific ‘PmrA box’ as (5ʹ HTTAAD-N_5_-HTTAAD), a finding later supported by subsequent studies [8,11].

Interestingly, sequencing of both clinical and laboratory-derived *A. baumannii* strains with elevated polymyxin resistance has revealed point mutations in the REC domain of PmrA. Given the critical role of PmrA in LOS modification, by increasing the expression of PEtN transferase PmrC, we sought to characterize how these mutations, D10N, M12I, I13M, G54E, and S119T (Table 1), impact various functions of PmrA’s receiver domain and upregulate the *pmrC* gene [12,13,14,15]. Mutations in response regulators often affect DNA-binding, oligomerization, and or phosphorylation/dephosphorylation rates [16,17,18]. Response regulator REC domains are conserved and have been studied extensively across many species [10,11,19,20,21]. The primary mechanism to propagate the phosphorylation signal is through perturbations of sidechains along what has been termed the ‘intra-protein communication pathway’ [9,10,14,15].

**Figure 1:**
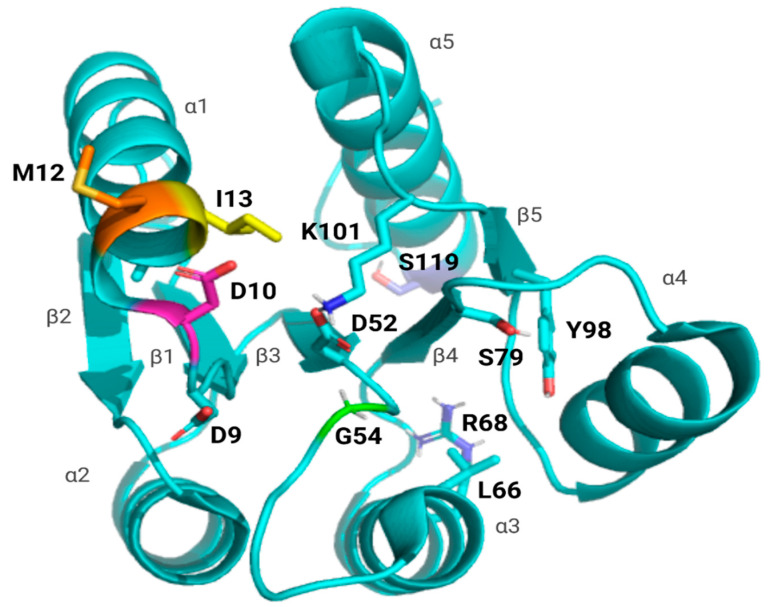
Map of WT PmrA REC domain showing mutations and key residues. Shown in cyan is the A chain of *A. baumannii* WT PmrA (PDB: 7M0S); each of the five β-strands and α-helices has been labeled. Key residues included within the intra-protein communication pathway are labeled and shown as sticks in cyan (D9, D52, L66, R68, S79, Y98, and K101). Mutations included in this investigation have been labeled and shown in various colors (D10 in pink, M12 in orange, I13 in yellow, G54 in green, and S119 in lilac).

This pathway includes several residues in specific regions of the REC domain (Figure 1). Each of these residues functions to either mediate the interaction with the kinase PmrB, coordinate with the incoming phosphate, or alter sidechain/local conformations to allow for dimerization and DNA-binding. D10 is a conserved residue within the PmrA aspartyl binding pocket, assisting in the coordination of the incoming phosphoryl group within the pocket [21,23,24,25]. Both M12 and I13 are located within the α1-helix, an area known to play a role in kinase recognition [26,27,28]. G54 is two residues away from the phosphorylated Asp residue (D52), residing close to key switch residues responsible for signal propagation [17,21]. Finally, S119 is located on the α5-helix, within the dimerization interface [29,30].

Through the evaluation of dimerization, DNA-binding affinity, phosphorylation, and the overall receiver domain structure, our goal was to determine how PmrA point mutations confer a resistant phenotype. These characteristics or features, if altered, can cause signal enhancement or disruption as well as modified protein–protein interactions, which culminate in diminished or enhanced gene expression. Our findings provide insight into how the REC point mutations may alter PmrA’s function to drive resistance. For the studied mutants, this occurs by interrupting the ability of PmrA to interact with its sensor kinase PmrB or by hindering phosphoryl group transfer from the kinase. Further understanding these molecular adaptations is crucial to developing effective countermeasures against the growing *A. baumannii* threat.

## 2. Materials and Methods

### 2.1. Protein Expression and Purification

Full-length PmrA and the N-terminal (PmrAN) from *A. baumannii* strain ATCC 19606 were expressed with an N-terminal His_6_ affinity tag using the pET28a expression vector. pET28a-PmrA (GenScript, Piscataway, NJ, USA) and pET28a-PmrAN mutants (D10N, M12I, I13M, G54E, S119T) (Genscript) were transformed into BL21 (DE3) *E. coli* cells. Overnight cultures were subcultured into 1 L LB and grown at 37 °C, 220 rpm to an optical density at 600 nm (OD_600_) of 0.7. Cultures were induced with 1 mM IPTG and grown overnight at 18 °C, 120 rpm. Cells were harvested, and pellets were resuspended in 80 mL lysis buffer containing 20 mM Tris, pH 7.5, 300 mM NaCl, and 5 mM imidazole. Cells were lysed by sonication, and the resulting clarified supernatant was loaded onto a Ni-NTA agarose column (Qiagen, Hilden, Germany) that was equilibrated with lysis buffer. Next, the protein was washed with 400 mL lysis buffer, followed by 400 mL 20 mM Tris pH 7.5, 1 M NaCl, and 5 mM imidazole. Protein was eluted using an imidazole gradient (5–200 mM) in lysis buffer; fractions were then pooled and concentrated to 150 mL using 10K Millipore spin columns. The His_6_ affinity tag was cleaved for 4 h at 26 °C, 75 rpm with the addition of 10 mg thrombin. The reaction was quenched with 20 μM 4-(2-Aminoethyl) benzenesulfonyl fluoride hydrochloride (AEBSF) for 30 min at room temperature. Protein was then dialyzed into 20 mM Tris, pH 7.5, 200 mM NaCl overnight.

Protein samples to be used for crystallography were not dialyzed initially but further purified using a HiPrep^TM^ 16/60 Sephacryl™ S-100 HR size exclusion column (Cytiva, Marlborough, MA, USA) equilibrated with 20 mM Tris, pH 7.5, and 400 mM NaCl. Fractions containing purified protein (based on SDS-PAGE) were combined and concentrated using Millipore 10 K spin columns (Burlington, MA, USA) according to the manufacturer’s protocol. Protein was stored at 4 °C for crystallography experiments.

### 2.2. Chemical Activation/Phosphorylation Mimic of Proteins with Beryllium Fluoride

Samples of 1 mg/mL purified protein were activated using beryllium fluoride (BeF_3_^−^). Protein was first chilled on ice for 30 min and then allowed to react with 7 mM MgCl_2_, 5 mM BeCl_2,_ and 35 mM NaF [20,31]. The solution was mixed and spun down at 4500 rpm for 15 min at 4 °C.

### 2.3. Native PAGE

To assess the oligomerization state of the PmrA and point mutations, Native PAGE was performed. Inactive protein samples were evaluated at 5 µM, 10 µM, and 20 µM in concentrations in 20 mM Tris pH 7.5, 200 mM NaCl, and 7 mM MgCl_2_. Reactions between PmrB, ATP, and PmrA were run with 40 μM PmrA and 20 μM PmrB. Samples were loaded on pre-chilled Novex 14% Tris-glycine acrylamide gels (Invitrogen, Carlsbad, CA, USA) along with a NativeMark protein standard (Invitrogen). Gels were run at 120 V with a 4 °C pre-chilled 1×Novex Tris-glycine buffer for 6 h. The gels were then stained with Coomassie and imaged on a Gel Doc EZ System (BioRad, Hercules, CA, USA). Figures were made using ImageLab 6.1

### 2.4. Ultra-Performance Liquid Chromatography-Size Exclusion Chromatography

UPLC-SEC samples were prepared as described in the Native-PAGE method for inactive protein. Samples were run at concentrations of 5 µM, 10 µM, and 20 µM. Prior to injection, all protein samples were centrifuged at 14,500 rpm for 10 min at 4 °C to remove any particulates. UPLC-SEC was performed using a Waters ACQUITY UPLC system (Waters, Milford, MA, USA) equipped with a Protein BEH SEC column (125Å, 1.7 µm, 4.6 mm × 150 mm, 1/pk). The mobile phase consisted of 20 mM Tris-HCl, pH 7.5, 200 mM NaCl, and 7 mM MgCl_2_, filtered through a 0.22 µm sterile filter and degassed using a bath sonicator for 10 min prior to use. Bio-Rad Gel Filtration Protein Standards were used for molecular weight estimation. Standards were rehydrated according to the manufacturer’s protocol in mobile phase buffer and filtered through a 0.22 µm sterile filter before injection. Samples were injected using the Waters ACQUITY UPLC system autosampler, with an injection volume of 10 µL. Experiments were performed at a flow rate of 0.3 mL/min for 10 min. Protein elution was monitored at 280 nm. Molecular weights of protein samples were estimated by constructing a calibration curve based on retention times of Bio-Rad protein standards.

### 2.5. Fluorescent Anisotropy DNA-Binding Assay

A 30 bp oligonucleotide sequence (5ʹHTTAAD-N_5_-HTTAAD) with a 5′-FAM (6-carboxyfluorescein) label was designed based on the *A. baumannii* PmrA box [8,11]. Equimolar concentrations of the 6FAM-labeled single-stranded DNA fragment from the *A. baumannii pmrC* promoter sequence (*AbpmrC*_30mer_FAM-F) and the complementary *AbpmrC*_30mer-R fragment (Integrated DNA Technologies, Inc., Coralville, IA, USA) were mixed and annealed by incubating at 94 °C for 2 min and allowed to cool gradually. Dilutions of the indicated protein concentrations were added to wells of black 96-well plates (BrandTech, Essex, CT, USA) containing 6′-FAM labeled dsDNA in 20 mM Tris, pH 7.5, 200 mM NaCl, and 2 mM MgCl_2_. The final reaction volume was 100 μL, and all assays were performed at room temperature. Fluorescent polarization was measured using a Synergy H1 Hybrid Multi-Mode microplate reader (BioTek, Winooski, VT, USA) and Gen5 software 3.02 (BioTek) at an excitation wavelength of 482 nm and an emission wavelength of 528 nm. To determine apparent-K_d_ values, data were analyzed using DynaFit 4 software (BioKinLtd., Minden, NV, USA) to fit a standard equilibrium mechanism, a + b ⇌ab [32]. Each experimental assay was performed with technical triplicates at least three times for each protein, both beryllium fluorinated and non-beryllium fluorinated.

### 2.6. Electrophoresis Mobility Shift Assay

DNA promoter fragments using the sequence determined by Palethorpe et al. (5ʹHTTAAD-N_5_-HTTAAD), the same sequence used for DNA anisotropy experiments without the 6FAM label [11]. Equimolar concentrations of single-stranded DNA (Integrated DNA Technologies, Inc.) were annealed by heating at 95 °C for 5 min in DNAase-free water, followed by slow cooling to room temperature. Binding reactions containing 1 μM DNA and indicated concentrations of protein were incubated for 5 min at room temperature before loading onto pre-chilled 8% TBE-acrylamide native gels. Samples were electrophoresed at 80 V in 1× TBE running buffer and stained with ethidium bromide for 5 min before visualizing bands. Gels were imaged and analyzed using ImageLab 6.1 software.

### 2.7. Radiolabeled (^32^P-ATP) Kinase Assay

A 20 μM solution of the cytosolic portion of *A. baumannii* histidine kinase PmrB (PmrBc) in 20 mM Tris-HCl at pH 7.5, 400 mM NaCl, and 5 mM MgCl_2_ was incubated with 15 μM ATP and 66.6 nM ^32^P-ATP (Revvity, Waltham, MA, USA) and allowed to autophosphorylate for 2 h at room temperature [33]. 40 μM of PmrA was then added to the reaction mixture. Then, 10 μL samples of reaction mixtures were taken at 0, 5, 10, 30, 120, and 360 min time points and mixed with 2 × sample loading dye (375 mM Tris, 6% SDS, 48% glycerol, 9% 14.7 M BME, 0.03% Bromophenol blue, and 10 mM EDTA). Samples were visualized by 14% SDS-PAGE gels. Gels containing phosphotransfer reactions to PmrA were run on gels for 45 min at 40 mAmps. The resulting gels were fixed in 30% methanol/10% acetic acid and exposed for 60 min to an Amersham storage phosphor screen. The screen was imaged on an Amersham Typhoon. Gels were then stained with Coomassie blue and imaged in a Gel Doc EZ System (BioRad). Gel images were analyzed in Cytiva ImageQuant^TM^ 10.2 (Marlborough, MA, USA).

### 2.8. X-Ray Crystallography

Crystals were grown using hanging drop vapor diffusion, and crystallization conditions are listed in Table 2. Crystals of PmrAN D10N, M12I, I13M, and S119T were grown at room temperature; PmrAN G54E crystals were grown at 18 °C. Crystals were harvested and cryo-cooled in liquid nitrogen. X-ray diffraction data were collected using the Advanced Photon Source (APS) beamline, 23-ID-D (GM/CA), at a wavelength of 1.03223 Å. Data was indexed, merged, and scaled using HKL-2000, and molecular replacement was achieved using PHENIX Phaser-MR [34,35]. Molecular replacement for PmrAN M12I, I13M, and S119T utilized *A. baumannii* PmrAN PDB ID 7M0S as a search model [11]. PmrAN D10N and G54E utilized AlphaFold2 to generate a monomer of PmrAN for molecular replacement [36]. Structures were refined in *Coot* and PHENIX.refine [37]. Structure figures were produced using PyMOL v3.0.

## 3. Results

### 3.1. A. baumannii PmrA and Mutations Bind the pmrC Promoter Sequence

**Figure 2:**
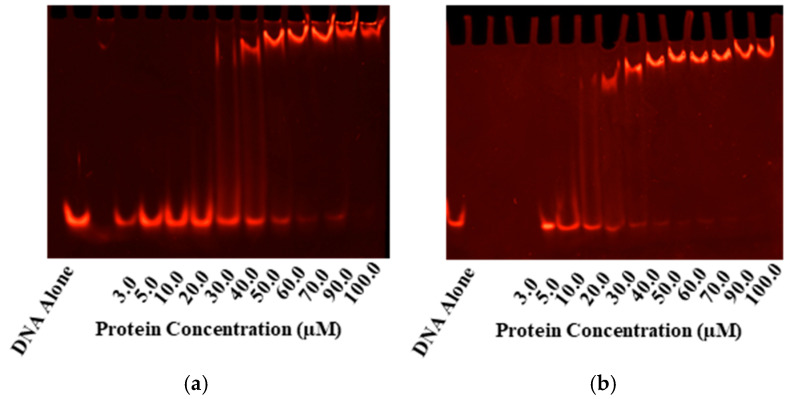

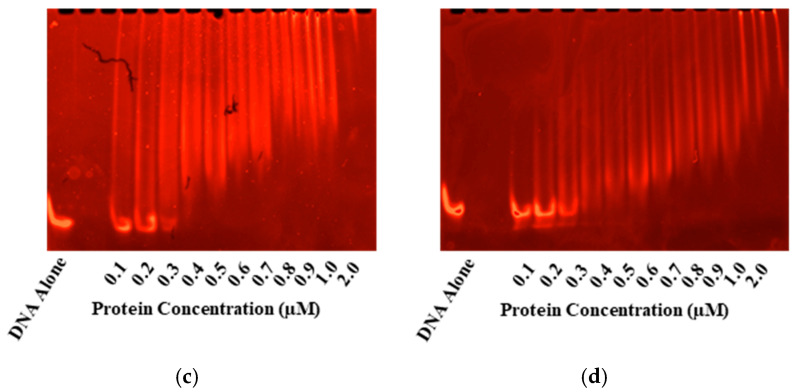
EMSAs for Gel-based Confirmation of DNA-binding. Electrophoretic mobility shifts are seen in the tandem repeat sequence with the consensus (-HTTAAD-N5-HTTAAD-) upon incubation with PmrA and its mutants in both the inactive (**a**,**b**) and BeF_3_^−^ activated PmrA (**c**,**d**). On the left, we have inactive and active WT PmrA (**a**,**c**). On the right, we have inactive and active PmrA mutant D10N (**b**,**d**). In each case, the ethidium bromide staining to track the movement of the 1 µM pmrC 30 bp promoter sequence is shown. Additional EMSAs may be found in Supplementary Figure S1.

**Figure 3:**
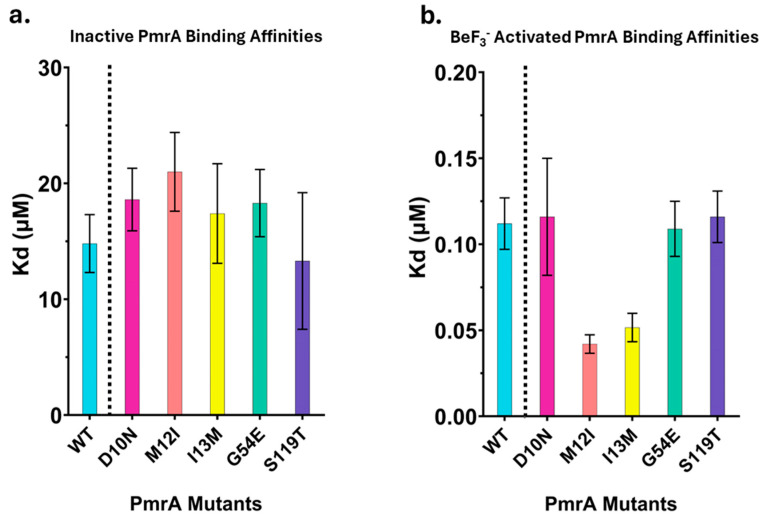
DNA-binding affinities of inactive and BeF_3_^−^ activated PmrA. Apparent Kd (μM) values from fluorescence anisotropy binding studies. Bars represent the average of at least three replicates; error bars represent standard deviation. WT in cyan, D10N in magenta, M12I in orange, I13M in yellow, G54E in green, and S119T in indigo. (**a**). Inactive PmrA samples. (**b**). BeF_3_^−^ activated PmrA samples.

PmrA binds a tandem repeating sequence (-HTTAAD-N_5_-HTTAAD-), which was used for EMSAs (Figure 2) and DNA anisotropy (Figure 3) [11]. EMSAs and fluorescence anisotropy confirmed that all mutants bound the *pmrC* promoter in both the inactive and the activated states. To activate PmrA, we employed beryllium fluorination, a reaction commonly used for response regulator activation [19,31]. The beryllium fluoride (BeF_3_^−^) mimics phosphorylation and provides a coordinate-covalent bond that allows for stable investigation of the active state.

Inactive WT PmrA binds with an apparent-Kd of 14.8 ± 2.5 µM. Inactive mutants bind the pmrC promoter with a similar affinity as the wild type (Figure 3a). As observed in previous investigations, activation with BeF_3_^−^ dramatically increases the binding affinity of WT PmrA (Figure 3b), apparent-Kd of 0.112 ± 0.015 µM (Table 3) [11]. Activated D10N, G54E, and S119T share a similar binding affinity to WT PmrA. M12I and I13M deviate from this trend slightly, binding with a two-fold higher affinity (Table 3, 0.0423 ± 0.0054 µM and 0.0516 ± 0.0083 µM, respectively) compared to WT. These results indicate that the PmrA mutations do not significantly impact the protein’s ability to bind the *pmrC* promoter sequence.

### 3.2. Oligomerization Evaluation of WT PmrA and Mutants

As dimerization is known to be integral in controlling response regulator activity, we sought to identify any effects caused by the point mutations. To determine the oligomerization states of PmrA, we employed native gels and UPLC-SEC to compare unphosphorylated (inactive) and phosphorylated PmrA mutants. The mobility of the PmrA point mutants was similar to that of the WT PmrA in the inactive state—monomeric species were observed for WT and mutant PmrA. Following phosphorylation, the WT and mutants dimerized. (Supplemental Figure S3). The unphosphorylated PmrA monomeric state was confirmed through SEC experiments (Supplemental Figure S4). The agreement in retention times for inactive PmrA and all the corresponding mutants (~4.4 min) suggests that the slight difference in electrophoretic mobility seen for D10N and G54E in the native gels was the result of charge disparity. The loss in mobility for D10N and the gain in mobility for G54E are due to the loss and gain of a negative charge in each mutant, respectively. The final calculated molecular weight for inactive PmrA was approximately 23 kDa, matching the theoretical molecular weight of monomeric PmrA, 24 kDa. The MW of phosphorylated WT PmrA and its corresponding mutants were observed using native gels, and its presence in the higher MW band corresponded to that of a dimer. Retention times and determined molecular weights are shown in Supplemental Table S1.

### 3.3. PmrA Mutants Exhibit Variations in Phosphorylation Rates

We next sought to examine the effects of the mutations on PmrA’s phosphorylation. To evaluate the rate of phosphorylation in vitro, we used a functional cytosolic truncation mutant of PmrA’s cognate histidine kinase PmrB, referred to as PmrBc. A qualitative examination of PmrA band intensity showed clear variation in phosphorylation rates between WT and mutants. We were unable to quantify both the rate of phosphorylation and dephosphorylation because PmrB, like most histidine kinases, exhibits phosphatase activity. Despite differences in phosphotransfer rates, once the phosphorylated population of PmrBc diminished, a rapid dephosphorylation of PmrA was seen. With no effective means of quenching phosphatase activity, the assessment of the results was limited to variations in the rate of phosphorylation for the WT and each PmrA mutant.

**Figure 4:**
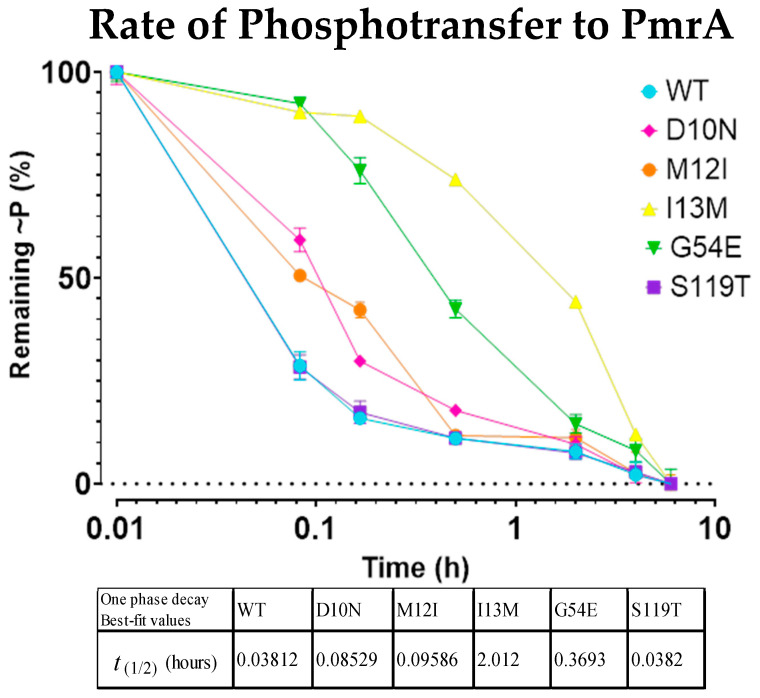
^32^P-bound PmrBc becomes depleted at different rates as it phosphorylates the WT PmrA and point mutations. Depletion rate of ^32^P~PmrBc as it interacts with and phosphorylates the WT PmrA and point mutations. Each mark indicates the standard deviation of the time point over the course of three replicates.

To assess these rates of phosphorylation, reactions were performed under ^32^P-ATP limiting conditions, and ^32^P~PmrBc populations were quantified in accordance with standard disappearance (substrate depletion) assays. I13M and G54E showed the clearest decrease in phosphorylation rates (Figure 4). While not as striking, D10N and M12I also had observable decreases in phosphoryl uptake. While we cannot entirely eliminate PmrBc’s phosphatase activity and its effect on the phosphorylation rate, any variation in PmrAB turnover between mutants and the WT would still have implications for gene regulation and resistance. 

### 3.4. Investigations into Structure Reveal Some Mutations May Enhance Constitutively Active Conformations

**Figure 5:**
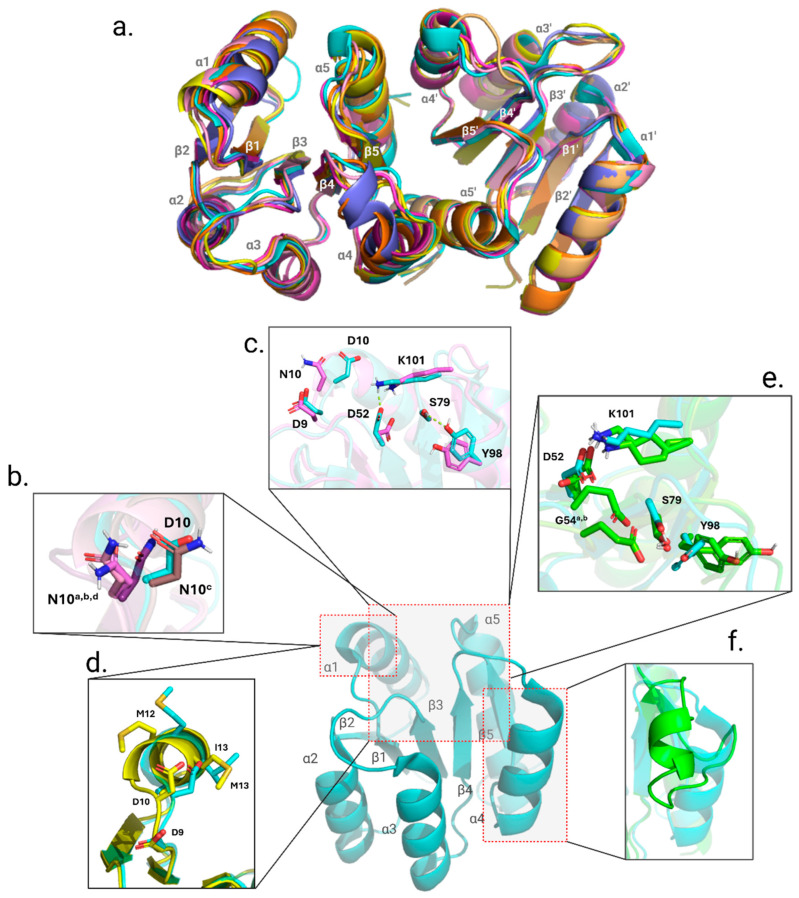
Alignment of secondary structure in all point mutations and variations in local conformation relevant to point mutation. The overall structure of the REC domain does not change. (**a**). The alignment of each mutant crystal structure (see text) to WT PmrAN (cyan). (**b**). Closeup of the a, b, c, and d chains in the D10N structure; the position of the D10N mutation sidechains in the a, b, and d chains is shown to highlight how they deviate from the orientation of the WT (cyan). (**c**). The departure of a typical orientation may affect the coordination of the aspartyl pocket, and the salt bridges between K101 and D52, as well as between S79 and Y98, are broken in the D10N structure (violet). (**d**). I13M (yellow) shows a departure from the typical orientation of both M12 and I13, both critical for kinase-response regulator communication. (**e**). The sidechains of G54E, chains A and B (green and chartreuse, respectively), both show evidence of sterically hindering the aspartyl pocket and potentially affecting the conformation of the switch residues. (**f**). The G54E structures showed the greatest deviation from the WT.

Finally, we probed for any structural alterations in the REC domain that may be brought on by the point mutations. Our phosphorylation studies demonstrated that D10N, M12I, I13M, and G54E mutants accept the activating phosphoryl group more slowly. Follow-up X-ray crystallography studies add context to the previous findings. As the mechanism for activation in PmrA and other response regulators is dependent on a domino effect of local conformational changes, observing sidechain repositioning of the intra-protein communication pathway may explain differences in phosphorylation acceptance rate. X-ray crystallography data were obtained for PmrA N-terminal domain (residues 1–124, PmrAN) mutants D10N, M12I, I13M, G54E, and S119T (Supplemental Table S2). Structurally, all mutants are similar to the starting model WT PmrAN (PDB ID 7M0S). Shown in Figure 5a is an alignment of each mutant crystal structure to highlight that there were no major conformational changes or alterations in secondary structure. The average Cα RMSD when aligned with WT ranged from 0.102 Å–1.19 Å (D10N chains in pink, Cα RMSD: 1.19 Å; M12I chains in orange, Cα RMSD: 0.146 Å; I13M dimer in yellow, Cα RMSD: 0.636 Å; and S119T in indigo, Cα RMSD: 0.102 Å). However, due to the relevance of sidechain positioning in response regulator activation, it is necessary to make note of local conformational perturbations.

While overall the secondary structure of PmrAN D10N remains greatly unchanged, the asymmetric unit (ASU) contains eight chains that are a combination of monomer chains and dimeric chains. Response regulators are well-known to crystallize as a dimer and often are solved with two chains in the ASU. The presence of eight chains is an indicator of an overall change in protein packing and relays that the dynamics of this mutant structure differ from those of the WT. As previously mentioned, the α1-helix is important for sensor kinase interaction and contains residues that are part of the conserved aspartyl pocket [38]. The α1-helix in PmrAN D10N shows that the mutated asparagine residue has altered the sidechain positioning in chains a, b, d, e, g, and h (Figure 5b,c). Also seen in the D10N crystal is the breaking of a salt bridge between D52 and K101 and a hydrogen bond between S79 and Y98 switch residues [21,23,39].

Effects from the point mutation within the α1-helix are also present in the dimeric PmrAN I13M crystal structure (Figure 5d). PmrAN I13M had two successfully solved crystal structures, one as a monomer and one as a dimer. Having the additional monomer structure aided in determining what deviations were due to the mutation and which were due to a conformational change from the monomer to dimer. The double methionines now present in the PmrAN I13M mutant resulted in a kink at the start of the α1-helix (M12 and the mutated M13, shown compared to the WT in Figure 5d). While the PmrAN M12I crystal structure did not show signs of any obvious deviations from WT, crystal packing in the ASU was unique, with four chains packing into the ASU, hinting at a change in protein dynamics and flexibility.

The greatest change from the WT PmrAN crystal can be observed in the PmrAN G54E structure (Figure 5e,f). PmrAN G54E crystallized with two chains in the ASU; however, they are monomeric in nature, lacking the typical signs of dimerization in response regulators. The α4-helix has become more disordered and broken from the typical orientation surrounding the β-strand core in the A-chain. Additionally, the site of the mutation is two residues away from the active site, D52. The substitution of the typical glycine with an asparagine residue adds a large, negatively charged sidechain that was previously small and nonpolar. In Figure 5e, the G54E sidechain in both chain A and chain B is between D52 and switch residue S79. In order for response regulators to achieve the active form, a domino effect of small conformational changes is necessary to carry the phosphorylation signal throughout the protein [21]. By having a larger negative sidechain between D52 and S79, this pathway is blocked and can result in difficulty accepting the phosphoryl group due to steric hindrance.

With appropriate resolution, crystallographic B-factors can reflect the amount of possible movement in a structure. To avoid bias in this analysis, it is important to use B-factor values from a nonsolvent-exposed region that is stable, such as the carbon backbone [40]. Within the PmrAN structures, the region of choice was the β-sheet core (β1, β3, β4). Overall, PmrAN D10N, M12I, I13M, and G54E had increased B-factor values, indicating an increase in overall flexibility and dynamics (Supplemental Figure S5). While most mutant PmrAN structures showed deviations from the WT structure to varying degrees, S119T was the exception. Interestingly, the B-factors for S119T were the only values that did not increase when compared to the WT.

Taken together, the data collected from our crystallographic studies add context to the phosphorylation studies. We suggest that the slowing of the phosphorylation acceptance rate can be attributed to two main causes. For PmrA M12I and I13M, we have determined that the mutations likely affect the kinase interaction α1-helix. Mutants D10N and G54E cause sidechain repositioning that disrupts the protein communication pathway by breaking necessary salt bridges, hydrogen bonds, or causing steric hindrance.

## 4. Discussion

The PmrAB system has been identified for its propensity to induce resistance to colistin, as well as other antibiotics, and its role in resistance has been studied extensively in various Gram-negative species, including *A. baumannii*, *Salmonella enterica* serovar Typhimurium, *Klebsiella pneumoniae*, *Pseudomonas aeruginosa*, and *Escherichia coli* [9,11,13,14,41,42,43,44,45,46,47,48,49,50,51,52]. Many mutations in PmrA have been documented, and their effects on outer membrane composition and MICs have been characterized [9,11,13,14,41,42,43,44,46,47,48,51]. This has provided insight into the features, at the organism level, that confer resistance to cationic antimicrobials like colistin, namely, altering the extent of lipid-A modifications. Despite the abundance of information regarding the effect of specific point mutants on the resistance of various pathogens and the specific features in these organisms that allow for resistance, there are few studies that have investigated how mutations in the receiver domain (N-terminus) are able to produce changes in gene expression. Some groups have examined the importance of the receiver domain. Lou et al. were successful in solving the crystal structure for *K. pneumonia* PmrA in complex with DNA and pursued additional NMR-based investigations to determine the importance of the REC-DBD interface in transcriptional activation [53]. While most of the point mutants studied were located in the DNA-binding domain (W181G, I220D, N188A, N196A, and R210A), N-terminal alanine screening (N43A, S46A, and N120A) demonstrated modest decreases in transcriptional activity in a reporter assay [53]. In a 2024 mechanistic and structural study on the *A. baumannii* PmrA, a crystal structure of the receiver domain highlighted the importance of the individual residues E8, D9, G54, K101, and L91, R111, R117, and R118 for coordinating the phosphorylation and dimerization events accordingly [9]. To follow up on the structural information, they determined that transcription for *pmrC* and *naxD* was diminished in the dimerization-deficient mutants as well as the phosphorylation-deficient mutant K101A [9]. In our previous work in 2022, structural, biochemical, and computational approaches were taken to identify critical features in the receiver domain of *A. baumannii* PmrA [11]. In this work, the N-terminal mutants I13M and P102R were probed to determine likely sources for the colistin resistance seen in *A. baumannii* harboring these mutations. The location of the I13M mutation and the increased conformational plasticity introduced by this mutation were thought to alter kinase interactions and stabilize the active-state to produce the modestly increased DNA-binding affinity seen [11]. Our current work evaluates several clinically and experimentally derived receiver domain mutations to determine the specific mechanism by which mutations in the receiver domain cause the upregulation of *pmrC*, leading to colistin-resistance in *A. baumannii*. While the work presented here focuses on *A. baumannii*, we believe that our conclusions would translate to other organisms possessing the PmrAB system; further investigation will be required to confirm this supposition.

The incentive for developing new antibiotics has declined due to high development and production costs outweighing profits [54]. The work here helps elucidate how mutations in the REC domain of the response regulator PmrA work to increase polymyxin resistance in *A. baumannii*. The goal of this study was to characterize the functional impact of these mutations by examining oligomerization, DNA-binding affinity, phosphorylation rate, and structure relative to WT PmrA. Oligomerization assays showed no significant differences between mutants or the wild type, regardless of the phosphorylation state. Similarly, DNA-binding assays confirmed that all mutants bind the *pmrC* promoter with affinities similar to WT PmrA regardless of the phosphorylation state. However, while the M12I and I13M mutants, upon BeF_3_^−^ activation, bound twice as tightly as WT, this two-fold increase is unlikely to be physiologically significant.

Most interestingly, the results from our phosphorylation experiments suggest that most of the PmrA mutants have a slower rate of phosphoryl uptake from PmrB compared to WT PmrA. Instead of tracking phosphorylation of PmrA, we opted to track the loss of the radio-labeled phosphoryl group in PmrB. This was due to the potential for spontaneous dephosphorylation of PmrA, and the phosphatase activity of its cognate histidine kinase PmrB.

Enhanced gene transcription may be accomplished despite slowed response regulator phosphorylation by extending the duration of the active, phosphorylated state. In some instances, sustained activation of the response regulator, rather than rapid and transient entrance and exit from the active state, can increase the total level of gene expression throughout the response period. This may be achieved through a process akin to kinetic proofreading, as well as through positive transcriptional feedback loops promoting response regulator production, or sustained gene expression [55,56]. This mechanism relies on a balance between the phosphorylating activity of a sensor kinase and its dephosphorylating phosphatase activity [57].

We propose a mechanism of PmrA activation based on the findings from our work. Over time, the accumulation of excess P~PmrA would likely be dephosphorylated before driving transcription [58]. Additionally, bacterial systems will often upregulate phosphatases in response to high phosphorylated protein levels, preventing excessive activation [59]. Considering these factors, overproduction and accumulation of P~PmrA would most likely be inefficient and energetically costly. A slower, more sustainable phosphorylation rate may optimize response activation while minimizing energy expenditure [60,61,62]. The reduced rate in phosphate uptake can be simplified into two main causes: either through alterations of key features within the active site or through disruption of the PmrB and PmrA recognition and interaction.

It is well-established that response regulator activation requires careful coordination within the Asp pocket (D9, D10, and D52). Acceptance of the phosphate generates a high-energy acyl phosphate and is necessary to drive the conformational cascade that enables activation. A divalent cation (Mg^2+^) and water molecules are required in the Asp pocket to accommodate the phosphoryl group, and to mediate interactions with the adjacent aspartate residues, D9 and D10 [18,21,23,39,63]. In addition to the three Asp residues that make up the pocket, a Lys residue (K101) forms a salt bridge with the phosphate. The key switch residues include a conserved Thr/Ser (S79) on β4 and a Phe/Tyr (Y98) on β5, which distinguish active from inactive conformations by the orientation of their sidechains [21,39]. Each residue in this pathway has a role to play in encouraging the active state of PmrA.

The upregulation of *pmrC*, leading to colistin resistance, by all the mutants, facilitated our initial hypothesis that an aspect of PmrA’s function would be enhanced and result in greater DNA-binding affinity. However, our findings did not align with our original hypothesis. Crystal structures revealed details of how the point mutations may slow phosphoryl acceptance. For the D10N mutation, we propose that the substitution of the conserved Asp residue with Asn reduces phosphorylation efficiency by disrupting the coordination of Mg^2+^, water molecules, and K101 by removing the negative charge needed to coordinate these active site components. Similarly, the G54E mutation results in significant slowing of phosphorylation. Located only two residues from the active site, the Glu sidechain appears to hinder hydrogen bonding between switch residue S79 and the phosphorylated active site. The impact of an additional negative charge in the active site has been observed in other response regulators, including CheY, and results in inhibition of autophosphorylation [16,17]. Since the G54E mutation is altering the net charge of the protein as observed through native gel experiments, the additional negative charge in the active site is a plausible explanation for the slowed phosphoryl uptake observed for G54E [64,65]. These data highlight that subtle changes in sidechain identity and orientation can have large downstream effects. Residues in the α1-helix interact with sensor kinases as well as phosphatases [23,66]. This implies that mutations in the α1-helix have the potential to alter interactions between PmrA and PmrB, a kinase and phosphatase [28,38,66]. Reducing PmrA-phosphatase binding would lead to reduced rates of PmrA dephosphorylation, prolonging the P~PmrA state [28,67].

In our previous work, as an alternate mechanism to enhanced DNA binding, we suggested the possibility that the PmrA::I13M mutation could affect interactions with RNA polymerase, and this could contribute to altered transcription of the *pmrCAB* operon [11]. It has also been proposed that interdomain dynamics between the receiver and DNA-binding domains aid PmrA in its interaction with RNAP holoenzyme to enhance gene transcription [53]. It is certainly possible that the resistant mutants studied here alter the interdomain dynamics to enhance RNAP function when compared to the WT. The S119T mutation did not significantly deviate from WT PmrA in any of the experiments included in this work. This mutation may employ one of the aforementioned mechanisms that would explain the increase in *pmrC* expression and ultimately polymyxin resistance.

Targeting the PmrAB two-component system remains an attractive avenue for therapeutic intervention in cases of polymyxin-resistant *A. baumannii*. In pursuit of effective intervention through this two-component system, our results reveal that impeding PmrA’s phosphorylation promotes sustained expression of the pmrC promoter, eliciting polymyxin resistance. These types of detail are key in designing effective and potent inhibitors; inadvertent slowing of the phosphorylation of PmrA by a prospective intervention could potentially lead to resistance.

## 5. Conclusions

Combating antibiotic resistance is a challenging feat due to the complexity and diversity of resistance mechanisms. This plight is emphasized when considering the nuances of a single system. The PmrAB system, responsible for colistin resistance in several virulent bacterial species, is a prime example of a complex system regulated by subtle changes. Our work here shows that single amino acid mutations in areas key to protein activity have understated effects that result in major downstream effects that ultimately encourage a resistance phenotype.

While our findings provide new insight into the molecular impact of PmrA mutations, much remains unknown about the precise regulatory mechanisms governing PmrA activation and transcriptional control. Our results, combined with prior studies showing increased *pmrC* expression and elevated colistin resistance in these mutants, support our hypothesis that altered PmrA regulation is central to *A. baumannii*’s ability to subvert polymyxin treatment. These findings also emphasize a broader point that combating antibiotic resistance requires a better understanding of the intricacies behind resistance mechanisms.

To conclude, in this present work, we provide strong evidence to suggest that point mutations in the receiver domain of the response regulator PmrA impact the ability of the protein to receive the phosphoryl group from PmrB. Contrary to our original expectations, the mutants investigated showed increased *pmrC* expression, an indication of enhanced PmrA function, but displayed no changes in PmrA DNA-binding affinity. This supports other models of PmrA-dependent transcriptional activation, which suggest that gene expression is not simply a matter of binding affinity [68]. Slowed phosphoryl uptake is supported by comprehensive structural data and results in colistin resistance by permitting a sustained activation signal.

## Figures and Tables

**Table 1:** MIC values of polymyxin-resistant strains of *A. baumannii*. Each strain had a point mutation in PmrA and an increase in the expression of PEtN transferase PmrC.

Polymyxin-Resistant *A. baumannii* Strains with Isolated Point Mutations and Respective Minimum Inhibitory Concentrations			
Mutations	Strain Derivation	MIC μg/mL	References
WT PmrA	ATCC 17978	0.5	Baumann P et al., 1968 [22]
D10N	Clinically	64	Palmieri, M. et al., 2020 [12]
M12I	Clinically	4	Arroyo, L. A. et al., 2011 [13]
I13M	Experimentally	32	Sun B, Liu H. et al., 2020 [14]
G54E	Experimentally	256	Oikonomou, O. et al., 2015 [15]
S119T	Clinically	16	Arroyo, L. A. et al., 2011 [13]

**Table 2:** Crystal Conditions for each PmrAN point mutation.

Point Mutation	Crystal Condition
D10N	0.1 M sodium citrate tribasic dihydrate, pH 5.6, 1.0 M NH_4_PO_4_
M12I	0.1M Bis-Tris pH 6.5, 0.2 M MgCl_2_, 25% PEG 3350
I13M (monomer)	0.2 M MgCl_2_, 0.1 M Tris pH 8.5, 30% PEG 4000
I13M (dimer)	0.2 M NH_4_Ac, 0.1 M NaCH_3_COO pH 4.6, 30% PEG 4000
G54E	0.2 M MgCl_2_, 0.1 M TRIS-HCl pH 8.5, 30% PEG 4000
S119T	0.2M NaCl, 0.1M BIS-TRIS pH 5.5, 25% PEG 3350

**Table 3:** Apparent Kds from DNA Anisotropy Experiments.

	Inactive		BeF_3_^−^ Activated	
	K_d_	SD	K_d_	SD
WT PmrA	14.8 µM	±2.5	0.112 µM	±0.015
D10N	18.6 µM	±2.7	0.116 µM	±0.034
M12I	21.0 µM	±3.4	0.042 µM	±0.005
I13M	17.4 µM	±4.3	0.051 µM	±0.008
G54E	18.3 µM	±2.9	0.109 µM	±0.016
S119T	13.3 µM	±5.9	0.116 µM	±0.015

## Data Availability

The original contributions presented in this study are included in the article/Supplementary Materials. Further inquiries can be directed to the corresponding author.

## Supplementary Materials

The following supporting information can be downloaded at https://www.mdpi.com/article/10.3390/microorganisms13112600/s1. Figure S1: EMSAs of pmrC promoter and PmrA mutants; Figure S2: Binding curves of fluorescence anisotropy experiments show affinity is relatively similar between most PmrA mutations in active and inactive states; Figure S3: Native gels show the oligomerization states do not vary greatly between WT and point mutations; Figure S4: UPLC-SEC chromatographs of PmrA oligomerization state; Figure S5: Normalized B-factor values of all collected PmrA REC structures; Table S1: Retention times and calculated molecular of inactive PmrA; Table S2: Data collection and refinement statistics (molecular replacement).
