# Supplementary material for: PmrA Mutations in Drug-Resistant Acinetobacter baumannii Affect Sensor Kinase-Response Regulator Interaction and Phosphotransfer
Source: Microorganisms. 2025 Nov 15;13(11):2600. doi: 10.3390/microorganisms13112600 (PMC12654582; doi:10.3390/microorganisms13112600)
Supplement: Supplementary file 1 [file microorganisms-13-02600-s001.zip › microorganisms-3953440-supplementary.pdf]

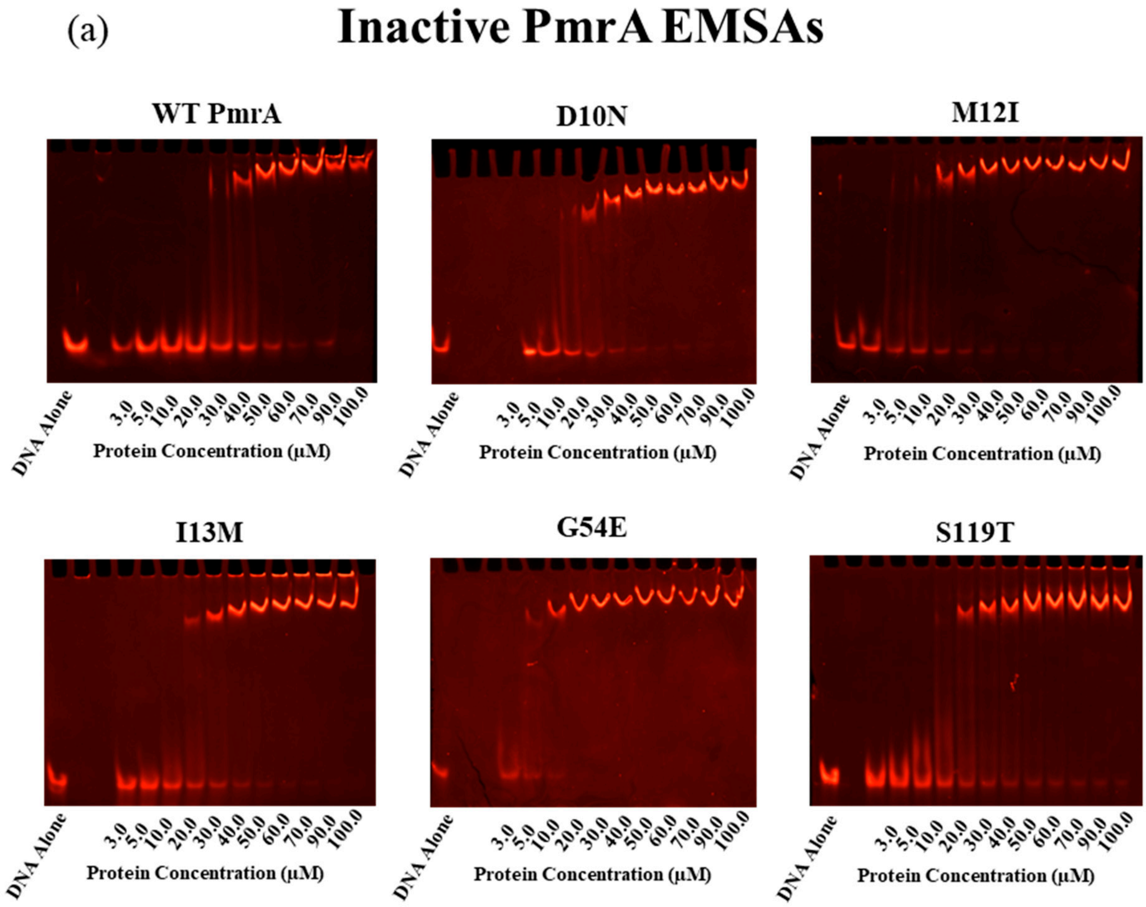
(a)
Inactive PmrA EMSAs
WT PmrA
D10N
M12I
I13M
G54E
S119T
DNA Alone
3.0 5.0 10.0 20.0 30.0 40.0 50.0 60.0 70.0 90.0 100.0
Protein Concentration (μM)

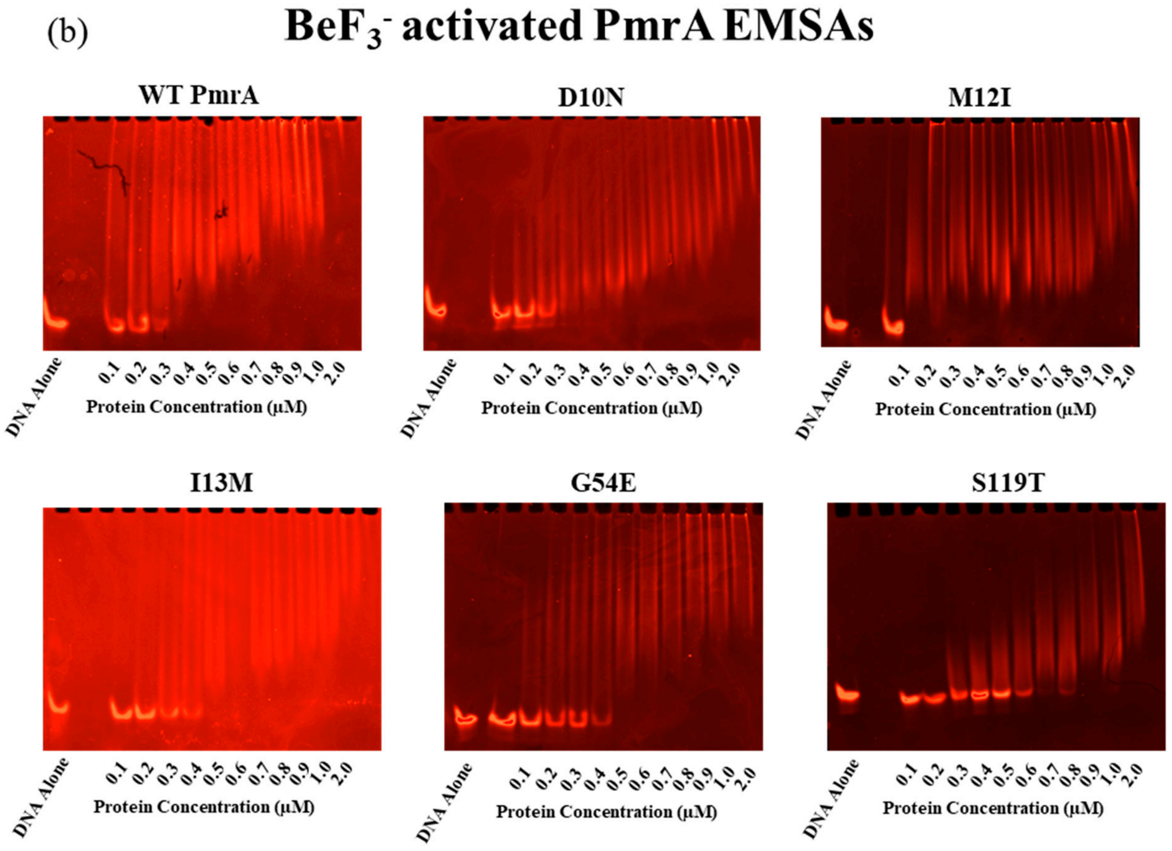

Supplemental Figure S1. EMSAs of *pmrC* promoter and PmrA mutants.

Binding of the WT PmrA and the PmrA point mutations was established using EMSAs. (a) EMSAs of inactive *A. baumannii* PmrA in increasing concentrations in the presence of 1 µM *pmrC* 30 bp promoter sequence (b) EMSAs of BeF_3^- *A. baumannii* PmrA in increasing concentrations in the presence of 1 µM *pmrC* 30 bp promoter sequence.

(a)

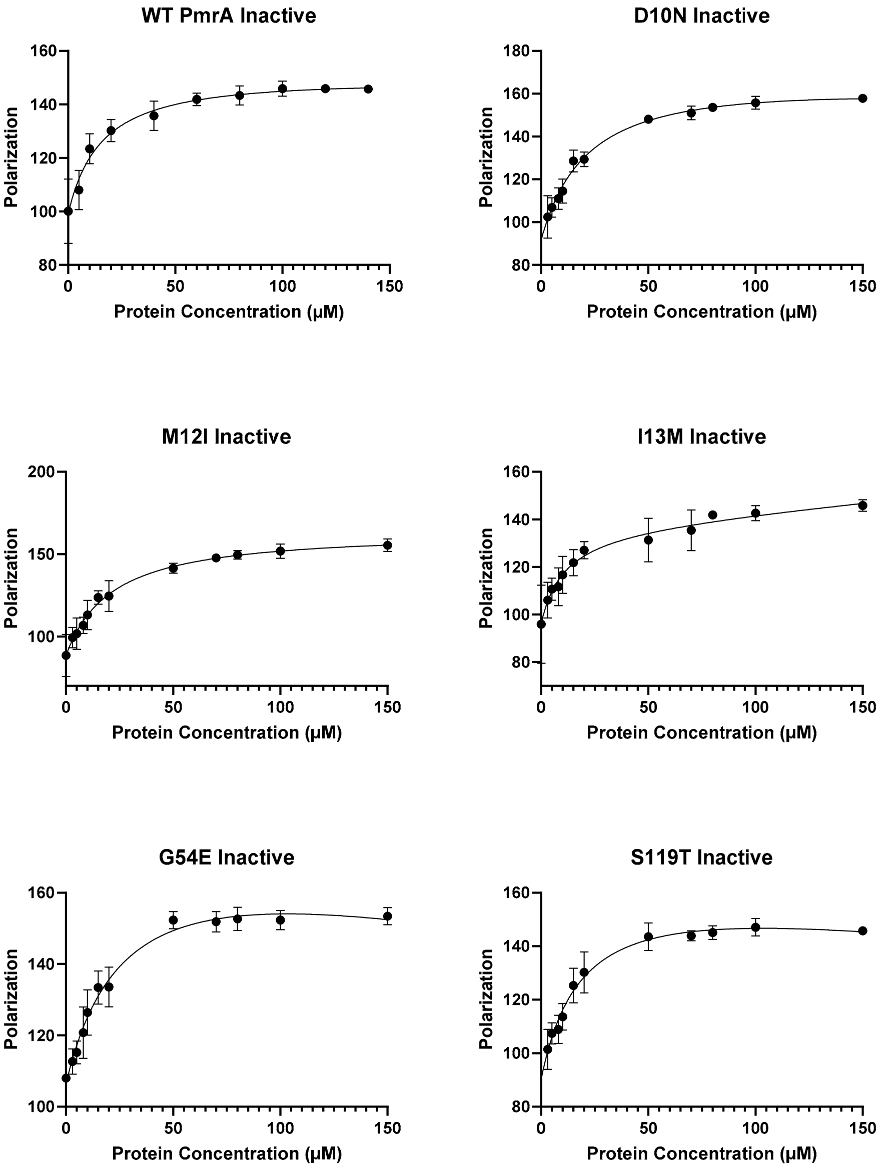
WT PmrA Inactive
D10N Inactive
M12I Inactive
I13M Inactive
G54E Inactive
S119T Inactive
Polarization
Protein Concentration (μM)
0
50
100
150
80
100
120
140
160
180
200

(b)

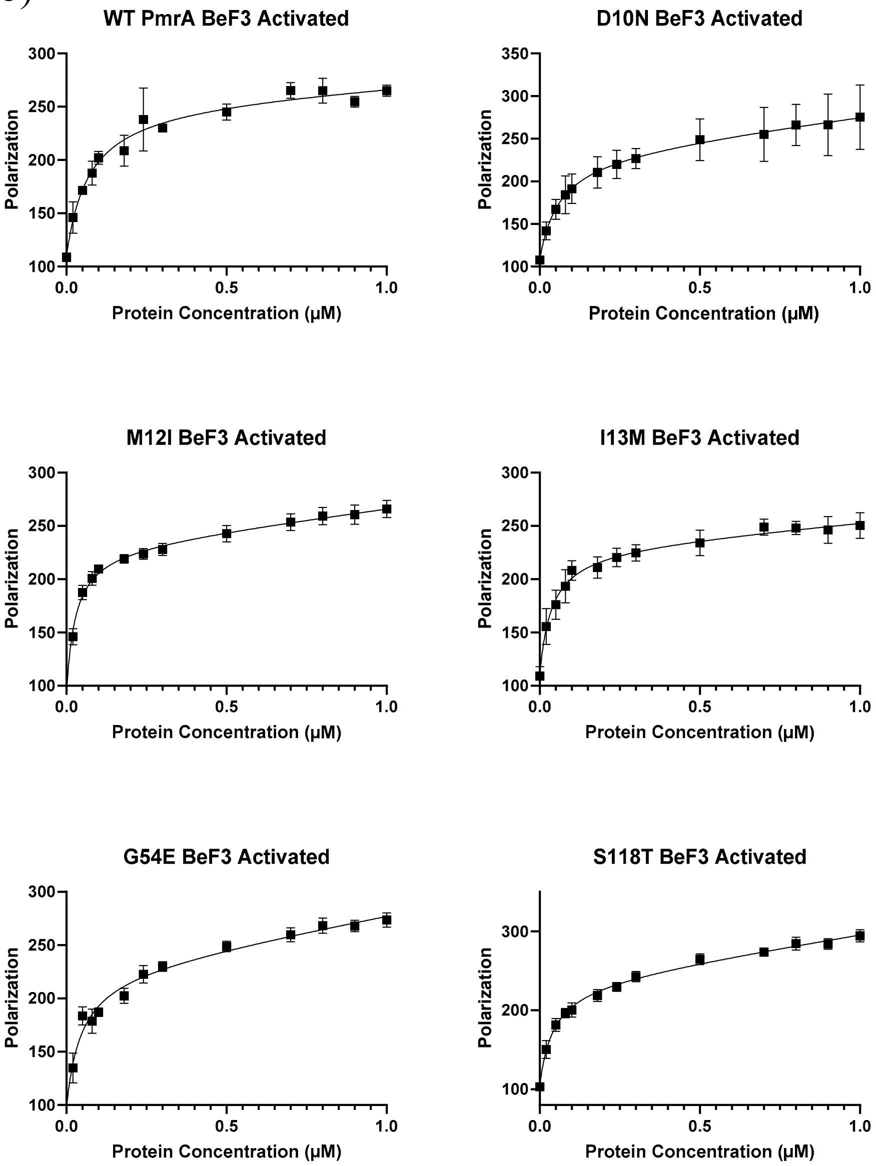

Supplemental Figure S2. Binding curves of fluorescence anisotropy experiments show affinity is relatively similar between most PmrA mutations in active and inactive states.

(a) 6FAM labeled *A. baumannii pmrC* promoter shows that the binding affinity of the PmrA point mutations is similar to the WT in the inactive protein. (b) 6FAM labeled *A. baumannii pmrC* promoter shows that the binding affinity of the PmrA point mutations is similar to the WT in the beryllium fluorinated active protein, however I13M and M12I binds the *pmrC* DNA with an affinity that is two times greater than WT.

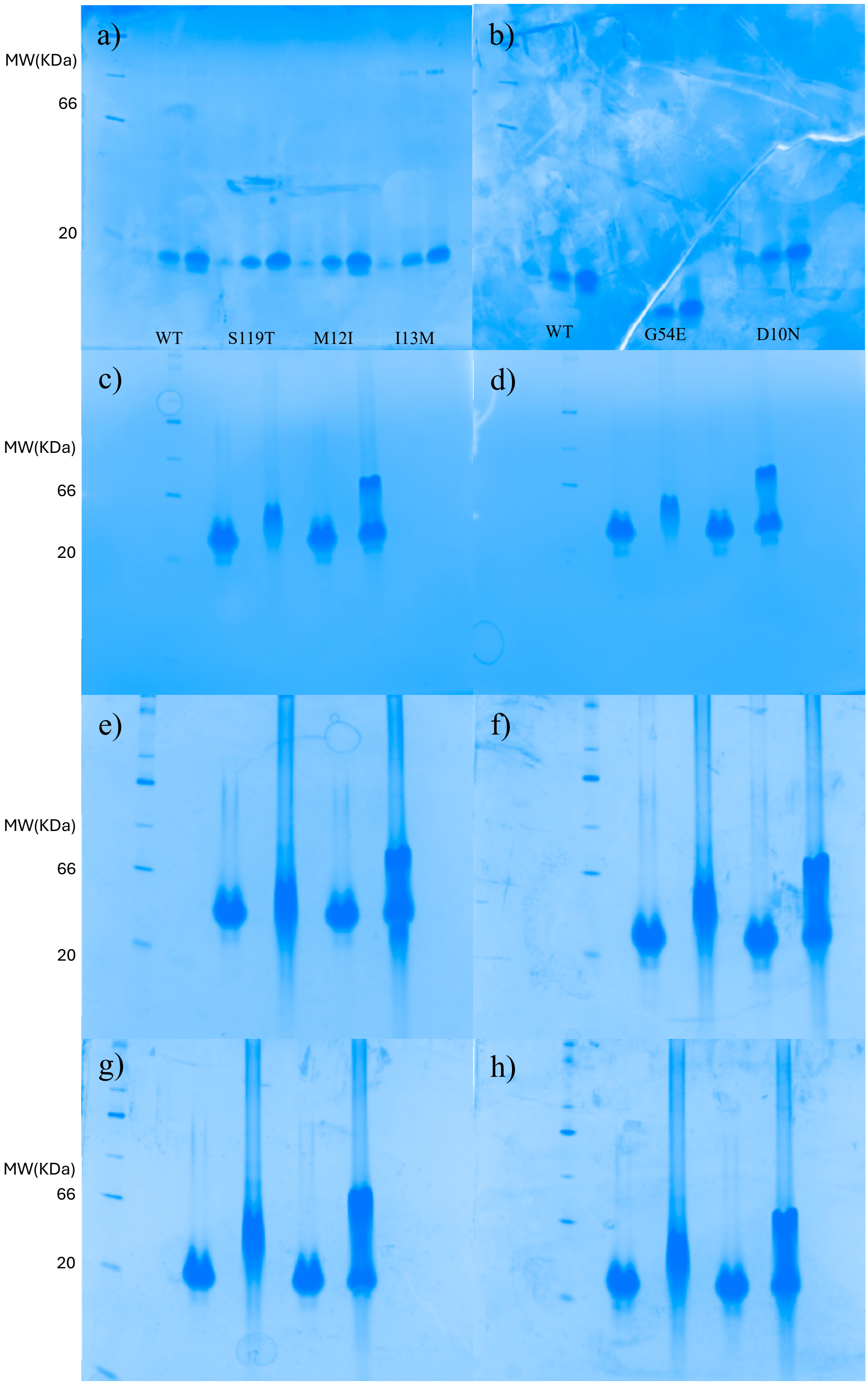

Supplemental Figure S3: Native gels show the oligomerization states do not vary greatly between WT and point mutations. (a, b) Native gels stained in Coomassie show there are no variations in oligomerization in the unactivated PmrA samples, shown going left from right in increasing concentrations (5 µM, 10 µM, 20 µM). (c-h) Activation of WT, M12I, D10N, I13M, G54E, S119T PmrA respectively. Lane 1, PmrA control, Lane 2 PmrB control, Lane 3 Pmra with ATP, and Lane 4 is the reaction of PmrA and PmrB in the presence of ATP showing a shift from the purely monomeric PmrA to a monomer-dimer equilibrium with a higher MW band corresponding to the approximate MW for the dimeric species.

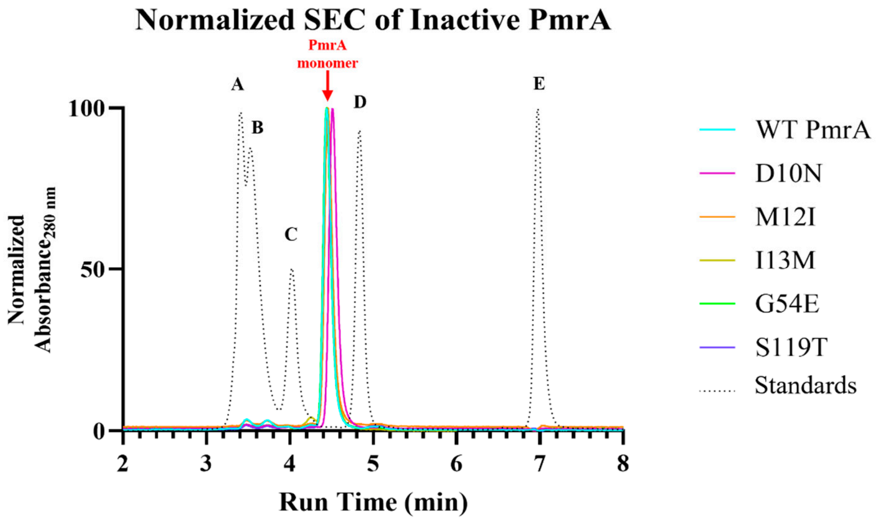

Supplemental Figure S4. UPLC-SEC chromatographs of PmrA oligomerization state.

Monomer peak highlighted in red. Molecular weights of protein samples were estimated by constructing a calibration curve based on retention times of Bio-Rad protein standard, show in the dotted black lines.

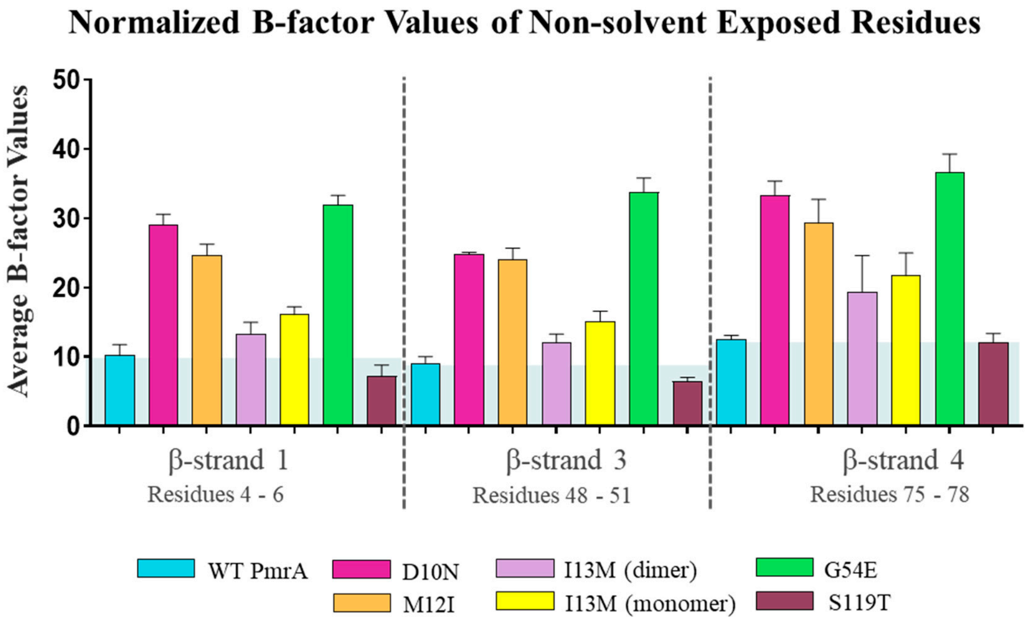

Supplemental Figure S5. Normalized B-factor values of all collected PmrA REC structures.
B-factor values were collected for each of the REC domain structures using PyMol software. This was done by selecting the β-strands in the center of the structure, those most stable and not in direct contact with the solvent (β1, β3, and β4). These data illustrated the increase in dynamics and flexibility in the mutant REC structures and compared them to the relative stability of the WT. All REC structures but one (S119T) followed this trend.

Supplemental Table S1. Retention times and calculated molecular of inactive PmrA.

Retention Times and Calculated Molecular Weights of Inactive PmrA Samples				
**PmrA Point Mutation**	**Retention Time (mins)**	**Average MW (kDa)**	**Std Dev**	**Theoretical Molecular Weight (kDa)**
WT PmrA	4.444	23.30	0.044	24
D10N	4.513	22.43	0.013	24
M12I	4.458	22.97	0.037	24
I13M	4.455	23.09	0.028	24
G54E	4.517	22.33	0.013	24
S119T	4.444	23.30	0.044	24

Supplementary Table S2. Data collection and refinement statistics (molecular replacement)

	D10N (9OF3)	M12I (9OF4)	I13M monomer (9OF5)	I13M dimer (9OF6)	G54E (9OF7)	S119T (9OF8)
**Data collection**						
Space group	$P4_12_12$	$P12_11$	$P2_12_12$	$P12_11$	$P12_11$	$P12_11$
Cell dimensions						
a, *b*, *c* (Å)	109.742, 109.742, 214.38	38.575, 59.45, 93.93	39.919, 96.688, 31.831	34.137, 35.517, 92.118	32.34, 110.634, 33.229	31.329, 38.890, 89.064
α, β, γ (°)	90, 90, 90	90, 90.068, 90	90, 90, 90	92.876, 90, 90	90, 102.915, 90	90, 92.728, 90
Resolution (Å)	50.00–2.67 (2.74–2.67)[a]	50.00–2.08 (2.15–2.08)[a]	50.00–1.65 (1.17–1.65)[a]	50.00–1.86 (1.91–1.86)[a]	50.00–1.99 (2.06–1.99)[a]	50.00–1.54 (1.60–1.54)[a]
$CC_{1/2}$	0.998 (0.964	0.986 (0.817)	1.00 (1.65)	0.983 (0.957)	1.00 (0.831)	0.993 (0.994)
R-merge	0.116 (0.390)	0.131 (0.444)	0.081 (0.623)	0.096 (0.279)	0.075 (0.488)	0.049 (0.096)
$I / \sigma I$	22.00 (3.50)	25.69 (4.72)	50.97 (3.88)	35.605 (10.262)	22.094 (1.812	50.365 (18.250)
Completeness (%)	97.17 (93.27)	96.06 (93.16)	99.82 (98.54)	99.8 (98.8)	95.68 (91.03)	97.47 (95.46)
Redundancy	10.7 (10.2)	4.3 (3.4)	7.9 (6.2)	6.0 (5.0)	5.5 (3.8)	6.4 (6.6)
**Refinement**						
Resolution (Å)	48.84–2.67	36.85–2.08	39.6–1.65	46.00–1.86	25.63–1.99	31.29–1.54
No. reflections	36906	24747	15543	18528	14843	31197
R_{work} / R_{free}	0.2245/0.2745	0.2223/0.2786	0.1785/0.2203	0.1873/0.2351	0.2164/0.2488	0.1727/0.1904
No. atoms	7602	4034	1124	2091	1961	2167
Protein	7586	3942	1002	2005	1927	2018
Water	14	90	121	86	32	149
Ligand	2	2	1	–	2	–
B-factors (Å^2)	33.96	32.52	21.58	25.6	42.83	13.36
Protein	33.99	32.54	20.75	25.46	42.92	12.86
Water	18.83	31.59	28.44	28.58	36.88	20.14
R.m.s. deviations						
Bond lengths (Å)	0.002	0.003	0.013	0.014	0.003	0.012
Bond angles (°)	0.466	0.543	1.24	1.211	0.563	1.22
Ramachandran favored (%)	98.06	97.53	97.54	99.19	98.28	98.77
Ramachandran allowed (%)	1.94	2.47	2.46	0.81	1.72	1.23

[a]Values in parenthesis correspond to the highest-resolution shell.
